# Augmented reality versus standard tests to assess cognition and function in early Alzheimer’s disease

**DOI:** 10.1038/s41746-023-00978-6

**Published:** 2023-12-18

**Authors:** Marijn Muurling, Casper de Boer, Srinivasan Vairavan, Robbert L. Harms, Antonella Santuccione Chadha, Ioannis Tarnanas, Estefania Vilarino Luis, Dorota Religa, Martha Therese Gjestsen, Samantha Galluzzi, Marta Ibarria Sala, Ivan Koychev, Lucrezia Hausner, Mara Gkioka, Dag Aarsland, Pieter Jelle Visser, Anna-Katharine Brem

**Affiliations:** 1 Alzheimer Center Amsterdam, Neurology, Vrije Universiteit Amsterdam, Amsterdam UMC location VUmc, Amsterdam, The Netherlands; 2 Amsterdam Neuroscience, Neurodegeneration, Amsterdam, The Netherlands; 3 Janssen Research & Development, LLC, 1125 Trenton Harbourton Road, Titusville, NJ 08560 USA; 4 Altoida Inc., Washington, DC USA; 5 Trinity College Dublin, Global Brain Health Institute – GHBI, Dublin, Ireland; 6 Centre de la mémoire, Université de Genève (UNIGE), Hôpitaux Universitaires de Genève, Geneva, Switzerland; 7 Center for Alzheimer Research, Department of Neurobiology, Care Sciences and Society (NVS), Karolinska Institutet, Stockholm, Sweden; 8 Centre for Age-related Medicine, Stavanger University Hospital, Stavanger, Norway; 9 Department of Clinical Medicine, University of Bergen, Bergen, Norway; 10 Laboratory Alzheimer’s Neuroimaging & Epidemiology, IRCCS Istituto Centro San Giovanni di Dio Fatebenefratelli, Brescia, Italy; 11 Ace Alzheimer Center Barcelona – Universitat Internacional de Catalunya, Barcelona, Spain; 12 Department of Psychiatry, University of Oxford, Oxford, UK; 13 Central Institute for Mental Health, Faculty Mannheim, University of Heidelberg, Heidelberg, Germany; 14 Alzheimer Hellas and Laboratory of Neurodegenerative Diseases, Center for Interdisciplinary Research and Innovation (CIRI – AUTh), Balkan Center, Aristotle University of Thessaloniki, Thessaloniki, Greece; 15 Institute of Psychiatry, Psychology and Neuroscience, King’s College London, London, UK; 16 Department of Psychiatry and Neuropsychology, School for Mental Health and Neuroscience, Maastricht University, Maastricht, The Netherlands; 17 University Hospital of Old Age Psychiatry, University of Bern, Bern, Switzerland

**Keywords:** Diagnostic markers, Alzheimer's disease, Alzheimer's disease, Preclinical research

## Abstract

Augmented reality (AR) apps, in which the virtual and real world are combined, can recreate instrumental activities of daily living (IADL) and are therefore promising to measure cognition needed for IADL in early Alzheimer’s disease (AD) both in the clinic and in the home settings. The primary aim of this study was to distinguish and classify healthy controls (HC) from participants with AD pathology in an early AD stage using an AR app. The secondary aims were to test the association of the app with clinical cognitive and functional tests and investigate the feasibility of at-home testing using AR. We furthermore investigated the test-retest reliability and potential learning effects of the task. The digital score from the AR app could significantly distinguish HC from preclinical AD (preAD) and prodromal AD (proAD), and preAD from proAD, both with in-clinic and at-home tests. For the classification of the proAD group, the digital score (AUC_clinic_visit_ = 0.84 [0.75–0.93], AUC_at_home_ = 0.77 [0.61–0.93]) was as good as the cognitive score (AUC = 0.85 [0.78–0.93]), while for classifying the preAD group, the digital score (AUC_clinic_visit_ = 0.66 [0.53–0.78], AUC_at_home_ = 0.76 [0.61–0.91]) was superior to the cognitive score (AUC = 0.55 [0.42–0.68]). In-clinic and at-home tests moderately correlated (rho = 0.57, *p* < 0.001). The digital score was associated with the clinical cognitive score (rho = 0.56, *p* < 0.001). No learning effects were found. Here we report the AR app distinguishes HC from otherwise healthy Aβ-positive individuals, both in the outpatient setting and at home, which is currently not possible with standard cognitive tests.

## Introduction

Alzheimer’s disease (AD) is a progressive neurodegenerative disease characterized by cognitive and functional decline. Many years before the clinical diagnosis, pathological amyloid beta (Aβ) and tau start to accumulate in the brain, in a stage called the preclinical phase^1^. The standard neuropsychological assessment used to support an AD diagnosis in the clinical setting usually includes a range of memory measures (episodic and visuospatial memory), executive functions (cognitive flexibility and inhibitory control), and complex attention (selective and divided attention). As complex instrumental activities of daily living (IADL), such as preparing a meal, managing finances, or using a technology such as a smartphone^2^ draw on a multitude of cognitive functions, the first signs of cognitive decline are likely reflected in subtle IADL impairment^3^. IADL informant ratings, such as the Amsterdam IADL questionnaire^4^, are therefore an important part of standard clinical routines. However, these cognitive and functional measures are, by definition, not sensitive enough to detect subtle changes in preclinical AD^1^, which would be beneficial for the identification of patients into and monitoring of patients during secondary prevention clinical trials. This is paramount, as only a trial can reveal whether there is a benefit to introducing screening for dementia within a population, or selecting at-risk populations. Moreover, not all participants have informants who know them well enough to answer questions on changes in IADL, especially cognitively unimpaired participants living alone.

Measuring cognition and IADL in early disease stages is a difficult task, as standard pen-and-paper tests are by definition not sensitive enough, and questionnaires rely on (informant) recall, which might not always reflect real-world situations. Real-world monitoring of cognition and IADL is not always possible, because it requires complicated setups, specialized personnel, and is time-consuming^5^. Augmented reality (AR) and virtual reality (VR) are new promising technologies that are helpful in measuring cognition and IADL because cognitive and IADL tasks using these techniques are comparable to real-life tasks. Moreover, the environment can easily be tailored to the needs of the patient and task^6^. VR was used in previous research on AD for diagnostics, screening, and interventions, recreating an IADL task^7,8^. AR merges the real world with virtual experiences, can easily be administered in the home setting on a smartphone or tablet, and is therefore cheaper and easier to use than VR. Further benefits of AR apps to measure cognition and IADL compared to conventional neuropsychological examination or questionnaires are that the task duration is significantly shorter, results are more objective, and tasks can be performed once per week or more without learning effects or increasing the workload of clinical personnel. Another advantage of AR apps is that the tasks are not necessarily linked to one specific IADL or a discrete cognitive domain but cover a range of day-to-day functions that are systematically involved and relevant for many IADLs^9^. Hence, measuring IADL-like activities with AR apps in the real world is thought to be highly promising to detect early AD, either in the context of patient selection for new disease-modifying therapies that are now available^10^, or to use as an endpoint in clinical trials. To the best of our knowledge, AR apps have not been used before to detect and monitor cognition and IADL in early AD^11^. Due to the aforementioned advantages, it is believed that AR apps are able to detect more subtle alterations in early AD compared to traditional tests, because apps allow for concomitantly assessing a multitude of features and provide more fine-grained and longitudinal information on behavioral changes.

The primary aim of this study is to explore the use of an AR app to measure cognition needed for IADL, and to distinguish and classify healthy older adults from participants with AD pathology in an early stage using this app and compare these classifications with classifications from standard clinical tests. The secondary aims are to test the association of the app with clinical cognitive tests and investigate the feasibility of at-home testing using AR. We furthermore investigate the test-retest reliability and potential learning effects of the task.

## Results

### Participants

No significant differences were found between groups for age, sex, and years of education (Table 1). By definition, MMSE was lowest in the prodromal AD (proAD) group, as participants were also grouped in accordance with the MMSE score. All participants performed the AR test in the clinic, and a subset performed the assessment at home. The total number of tests at home did not differ between groups.

**Table 1:** Demographic characteristics per study group.

	Healthy control	Preclinical AD	Prodromal AD	*p* value
*N*	57	27	37	
Age	68 (7)	71 (5)	70 (8)	0.12
Years of education	15 (3)	15 (3)	15 (5)	0.69
Male, *n* (%)	26 (46%)	10 (37%)	24 (65%)	0.06
Site, *n* (%)				
- Amsterdam	20 (35%)	12 (44%)	10 27%)	
- London	5 (9%)	0 (0%)	3 (8%)	
- Oxford	5 (9%)	0 (0%)	1 (0%)	
- Stockholm	0 (0%)	2 (7%)	6 (16%)	
- Thessaloniki	5 (9%)	0 (0%)	3 (8%)	
- Ljubljana	2 (4%)	1 (4%)	1 (0%)	
- Lisbon	5 (9%)	0 (0%)	2 (5%)	
- Brescia	4 (7%)	0 (0%)	3 (5%)	
- Geneva	2 (4%)	5 (19%)	4 (8%)	
- Mannheim	0 (0%)	0 (0%)	3 (8%)	
- Barcelona	4 (7%)	0 (0%)	2 (3%)	
- Stavanger	5 (9%)	7 (26%)	4 (11%)	
MMSE	29 (1)	29 (1)	27 (2)	<0.001
Aβ available, *n* (%)	43 (75%)	27 (100%)	37 (100%)	
DSST score	49 (11)	46 (10)	35 (13)	<0.001
Verbal fluency score	64 (16)	66 (16)	53 (19)	0.003
Word list recall score, %	70 (20)	74 (20)	35 (29)	<0.001
Boston naming test score, %	94 (6)	92 (7)	87 (13	0.001
Rey drawing score	34 (2)	34 (2)	31 (7)	<0.001
Rey recall score	21 (7)	19 (6)	13 (8)	<0.001
Cognitive score	−0.05 (0.67)	−0.16 (0.58)	−1.50 (1.38)	<0.001
A-IADL score	68.4 (2.6)	66.8 (3.0)	60.8 (6.5)	<0.001
Digital score	62.2 (21.3)	50.7 (21.6)	33.2 (20.0)	<0.001

Numbers show mean (SD), unless specified otherwise. The different sites used their own local language for the tests. *P* values are from ANOVAs or *χ*^2^ tests where appropriate, uncorrected for any covariates.

*Aβ* amyloid beta, *AD* Alzheimer’s disease, *A-IADL* Amsterdam instrumental activities of daily living, *DSST* Digit Symbol Substitution Test, *MMSE* Mini-Mental State Examination.

### Discrimination of groups using the AR app

**Fig. 1:**
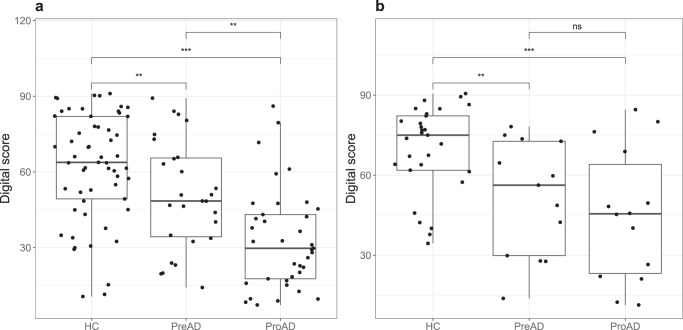
Digital scores from the AR app presented per study group. **a** In-clinic test (*N* = 121). **b** At-home test (*N* = 56). Each dot represents the digital score of one participant. The box represents the lower and upper quartiles with the center line the median, and the whiskers represent the minimum and maximum score. Group differences were tested using a linear model, corrected for app version and site. **p < 0.01, ***p < 0.001, ns indicates not significant. HC healthy control, preAD preclinical AD, proAD prodromal AD.

When comparing the three study groups on the digital score resulting from the in-clinic AR task (Fig. 1a), the proAD group scored significantly lower compared to the healthy controls (HC, *β* ± SE = −32.3 ± 4.9, *p* < 0.001) and preclinical AD (preAD) group (*β* ± SE = −16.2 ± 5.4, *p* = 0.003). The preAD group also scored lower than the HC group (*β* ± SE = −16.1 ± 5.3, *p* = 0.003). When comparing the three study groups on the digital score using the first at-home test, which included a smaller sample size (Fig. 1b), the HC group scored significantly higher than the preAD (*β* ± SE = −20.6 ± 6.9, *p* = 0.005) and proAD group (*β* ± SE = −25.9 ± 7.0, *p* < 0.001) as well, but there was no significant difference between the preAD and proAD groups (*β* ± SE = −5.3 ± 7.8, *p* = 0.50). After stratification for sex, the differences in the whole cohort were observed for females, but the HC–preAD difference was not found in males (Supplementary Fig. 1).

### Classification of groups using the AR app and clinical tests

**Fig. 2:**
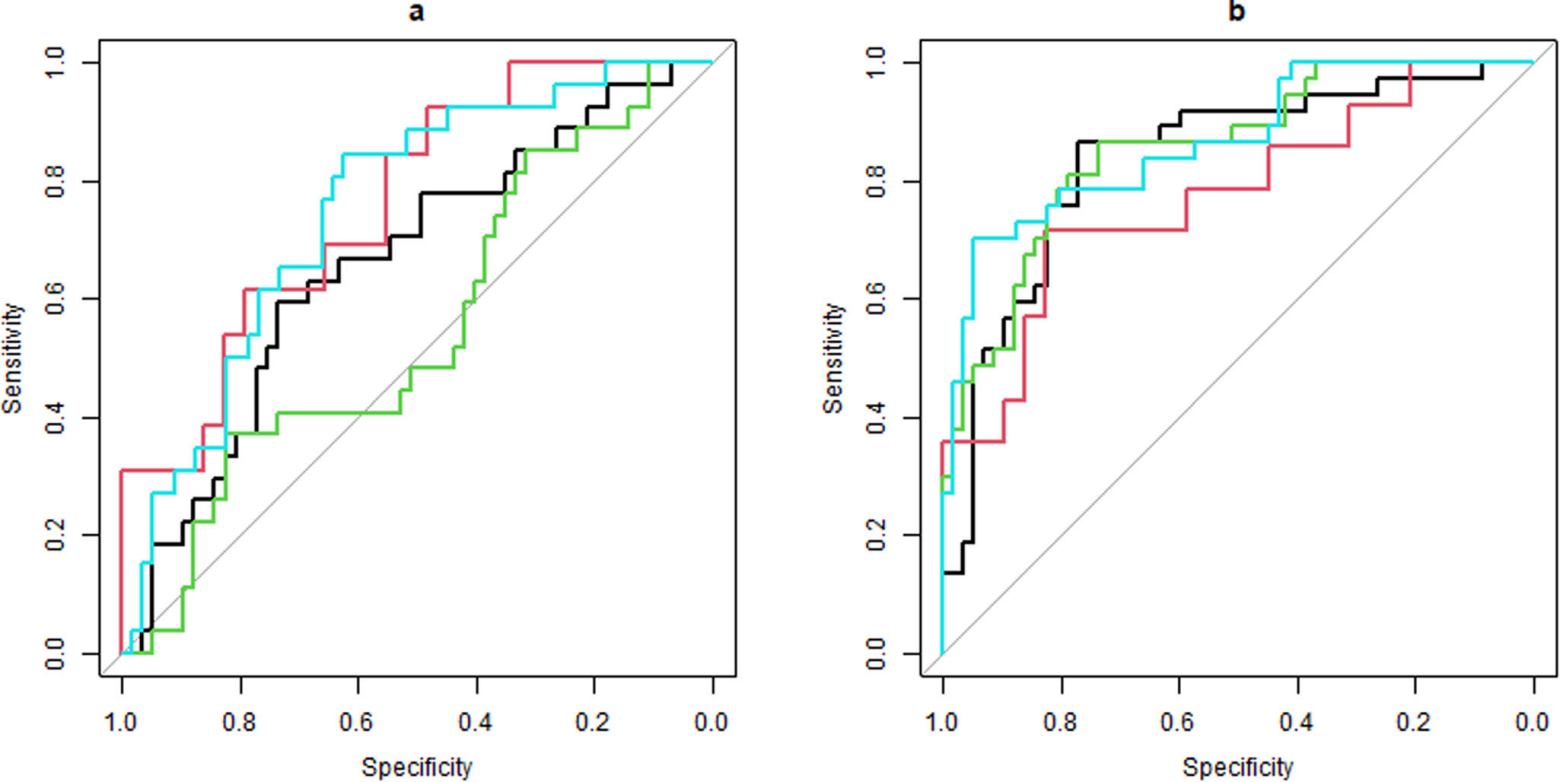
ROC curves for the digital in-clinic, digital at-home, cognitive, and A-IADL score. **a** Classification of healthy controls relative to preclinical AD. **b** Classification of healthy controls relative to prodromal AD. Black line shows digital in-clinic test curve, red line shows digital at-home test curve, green lines shows cognitive score curve, and light blue line shows A-IADL score curve. A-IADL Amsterdam instrumental activities of daily living, HC healthy control, PreAD Preclinical AD, ProAD Prodromal AD.

For the classification of the proAD group from the HC group (Fig. 2, right panel), the digital in-clinic score (ROC-AUC = 0.84 [0.75–0.93], PR-AUC = 0.87), digital at-home test (ROC-AUC = 0.77 [0.61–0.93], PR-AUC = 0.86), and the A-IADL score (ROC-AUC = 0.86 [0.79–0.94], PR-AUC = 0.90) were statistically (*p* > 0.05) as good as the cognitive score (ROC-AUC = 0.85 [0.78–0.93], PR-AUC = 0.90). For classifying the preAD group from the HC group (Fig. 2, left panel), the digital in-clinic score (ROC-AUC = 0.66 [0.53–0.78], PR-AUC = 0.80), digital at-home score (ROC-AUC = 0.76 [0.61–0.91], PR-AUC = 0.89), and A-IADL score (ROC-AUC = 0.75 [0.64–0.86], PR-AUC = 0.87) performed better than the cognitive score (ROC-AUC = 0.55 [0.42–0.68], PR-AUC = 0.74), although only the AUC of the digital at-home score and A-IADL score were significantly superior to the cognitive score (*p* = 0.02 and *p* = 0.008 respectively). When using the subgroup of participants who completed at least three at-home tests, results were similar (Supplementary material). PR curves are shown in Supplementary Fig. 2.

### Association between clinical tests and AR app

**Fig. 3:**
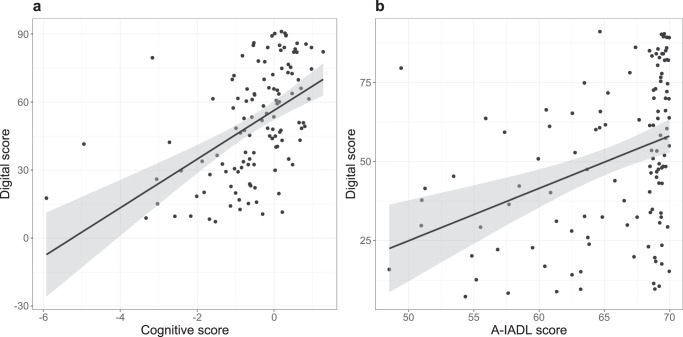
Association of digital score with standard tests. **a** Association of digital score (in-clinic) with the cognitive score (Spearman’s rho = 0.56, *p* < 0.001). **b** Association of digital score (in-clinic) with the A-IADL score (Spearman’s rho = 0.43, *p* < 0.001). Each dot represents the scores of one participant. The black solid line represents the correlation line with the 95% confidence interval in gray. A-IADL Amsterdam instrumental activities of daily living.

The digital in-clinic score was significantly and positively correlated with the cognitive score (Spearman’s rho = 0.56, *p* < 0.001) (Fig. 3a) and the A-IADL score (Spearman’s rho = 0.43, *p* < 0.001) (Fig. 3b). Significant correlations were also found for the separate cognitive tests: DSST (rho = 0.45, *p* < 0.001), verbal fluency (rho = 0.27, *p* < 0.003), word list learning recall (rho = 0.56, *p* < 0.001), Boston naming test (rho = 0.37, *p* < 0.001), Rey drawing score (rho = 0.19, *p* = 0.04), and Rey recall score (rho = 0.43, *p* < 0.001).

### Feasibility of the AR app at home

**Fig. 4:**
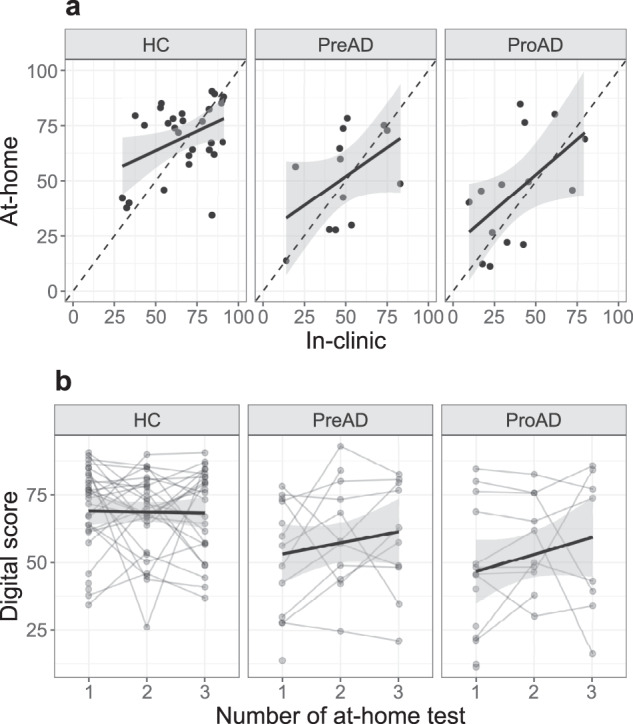
Within-subject associations. **a** Correlation between in-clinic and at-home tests (HC: rho = 0.34, *p* = 0.07; preAD: rho = 0.54, *p* = 0.06; proAD: rho = 0.58, *p* = 0.03). Each dot represents the digital score from one participant. The black dashed line is the rho = 1 line. The black solid line shows the correlation line with the 95% confidence interval in gray. **b** Change of digital score over time. Each line represents one participant. The black solid line represents the average change over time with the 95% confidence interval in gray. Prodromal AD participants showed overall lower scores, but no learning effects were seen. HC healthy control, PreAD Preclinical AD, ProAD Prodromal AD.

There was a significant moderate correlation between the test in the clinic and the first test at home (Spearman’s rho = 0.57, *p* < 0.001). When calculating three separate correlations per study group (Fig. 4a), correlation coefficients increased with disease stage (HC: rho = 0.34, *p* = 0.07; preAD: rho = 0.54, *p* = 0.06; proAD: rho = 0.58, *p* = 0.03).

### Test-retest reliability

While the in-clinic assessments consisted of one administration, a total of 43 participants had at least 3 at-home tests with reliable data (no technical issues reported during the bi-weekly phone calls) to examine test-retest reliability. Test-retest reliability for the first three at-home tests was poor with ICC = 0.48 and 95% confidence interval (CI) = 0.29–0.65. When stratifying for phone type, the ICC for Android users was higher (ICC = 0.70 [0.43–0.89]) than for iOS users (ICC = 0.33 [0.11–0.57]).

### Learning effect

A total of 43 participants had at least 3 at-home tests with reliable data to examine learning effects, but participants who had at least 1 at-home test were included in the linear model. The interaction of group and test number was not significant (preAD*test number: *β* ± SE = 4.9 ± 3.5, *p* = 0.17, proAD*test number: *β* ± SE = 3.7 ± 3.7, *p* = 0.32) showing no increase or decrease of the digital score over time (Fig. 4b).

## Discussion

The aims of this study were to test the ability of an AR spatial-navigation app to distinguish HC participants from early AD participants, test the association with clinical cognitive tests and the feasibility of at-home testing. Specifically, we were interested in detecting differences between HC and preAD, which is currently not possible to achieve with standard tests and is of imminent importance given the recent advances in disease-modifying treatments. We also investigated test-retest reliability and potential learning effects. We found that HC participants could be differentiated from both preAD and proAD participants, both in the clinic and at home, which was not possible using a standard neuropsychological assessment for the preAD group. In-clinic and at-home tests showed a significant but moderate association, no learning effects were seen, but test-retest reliability for at-home iOS users was subpar.

The digital score performed equally well as the cognitive score and A-IADL score in discriminating cognitively normal (i.e., HC and preAD) participants from cognitively impaired (i.e., proAD) participants, indicating that the discriminative power of the AR app was as good as conventional neuropsychological tests and questionnaires. This was confirmed by the good performance of the sensitivity-specificity analysis (AUC = 0.84). The most important challenge was to differentiate HC participants from preAD participants, which is currently difficult using conventional pen-and-paper tests. To our knowledge, this is the first study using AR to distinguish preAD from HC. Our models showed that the AR app could significantly distinguish HC from preAD, while the neuropsychological battery failed to do so. These findings are promising to encourage future research to further develop this method. Since apps can make use of all sensors a device provides (e.g., accelerometers, gyroscopes, magnetometers, microphone, touch screen), it was expected that they can detect more subtle changes in preAD than conventional pen-and-paper tests. Through these means, digital technology can enable programs aimed at the secondary prevention of dementia through the identification of at-risk individuals. Still, the AUC of the digital score to distinguish preAD from HC was only moderate (AUC = 0.66), but this was expected since the digital score was based on a machine learning model trained to distinguish cognitively impaired from cognitively normal individuals (proAD vs. HC). Further research should therefore focus on developing specific machine learning models for the digital score to distinguish preAD from HC with even greater accuracy.

Important to note is that the AR tasks measure a broader concept of cognition needed for IADL. This is confirmed by the association between the digital score and the cognitive score, which includes tests from multiple cognitive domains as well as associations with these cognitive subdomains. So, although there might not be a direct association between established, single IADL functions and the AR task requirements in this study, the app aims to assess, in a standardized way, combinations of relevant features that are necessary for a range of IADL. Notably, with the app we are able to capture features that cannot be assessed with standard tests to date.

It was striking that the digital scores of the cognitively normal groups showed a high variation, some even lower than the mean of the proAD group (Fig. 1). A possible explanation for this result is the high heterogeneity within groups, or that the digital score captures more subtle differences than conventional assessments, particularly at very early disease stages. Supporting this hypothesis is the sex difference we found, whereby the AR app could distinguish preAD from HC in females but not in males. Females are known to have more advanced tau spread in the brain compared to males for a given clinical stage^12^, different disease manifestation and disease trajectories^13^, and seem to benefit less from recently approved disease-modifying treatments^14^. The AR app could measure more sensitively, and therefore could have detected the more advanced pathological disease state of females in the preAD group opposed to males. Indeed, a previous study has shown that a biological sex classifier that was built on digital biomarker features captured using the AR app achieved an ROC-AUC of 75% for predicting biological sex in healthy individuals, indicating that there are differences in neurocognitive performance signatures between males and females^15^. With additional data, particularly from participants with known AD biomarker status, the model for the digital score could be enhanced to improve the predictions.

A clear advantage of using the AR app over standard neuropsychological tests was the better discrimination of diagnostic groups, while the time needed to execute the short neuropsychological assessment used in this study was similar to the time needed for the AR app when including the explanations and practice rounds, which was approximately 20 min. Another advantage of the AR tasks is that these tasks can be performed at home, without the need of a trained neuropsychologist. If used in clinical trials, at-home testing would lower participant burden and trial costs, while in clinical practice, at-home testing would be valuable for easy and low-cost at-home screening for AD. Now that disease-modifying therapies have become available^10^, early detection of AD pathology becomes more important, as amyloid positivity is a requisite for receiving anti-amyloid immunotherapy. The discriminative ability of the HC-preAD groups of the at-home tests was just as good as in-clinic, supported by good correlation of results between environments (rho = 0.57), emphasizing the advantage of at-home use. No difference between preAD and proAD was found using the at-home tests, probably due to a reduced sample size with participants who were cognitively less impaired, reducing the differences between groups. Unexpectedly, this correlation was somewhat lower in cognitively normal groups, and improved slightly in the proAD group. It seems that cognitively normal groups performed better at home than in the clinic, which could be related to the fact that the richer and well-known home environment poses less demands on memory and participants feel less observed.

The subpar test-retest reliability, particularly for iOS users, was unexpected. Possible explanations are the use of different versions of the AR app (version 1 and 2, with version 2 being more reliable for iOS users), the low number of participants using the app at least three times at home (*N* = 43), different phone models used, and possible help of a study partner for some but not all tests, although specifically instructed not to. To confirm these results, further research should perform the tests in a controlled environment with the same phone type and AR app version.

Notably, only 59% of the proAD participants used the app at home, as opposed to 72% and 70% of the HCs and preAD participants, respectively. Moreover, the participants who had to be excluded because of technical issues were highest in the proAD group. The reason for this could be purely technical, i.e., they did not have smartphones that were compatible with the app, or technical difficulties arose due to their cognitive impairment. The first reason could easily be solved by provisioning devices instead of “bring-your-own-device”, which would decrease the selection bias^16^ and generate more controlled outcomes by utilizing standardized hardware and software^17^. The latter reason for more technical issues in the proAD group would, however, be a concern because this implies that these types of apps are not useful in, for example, clinical trials in this population. Our data suggests that there were more participant-reported problems with the AR app in the proAD group than in the other groups, which suggests that cognitive impairment could be affecting their ability to perform the AR tasks at home.

Our findings are relevant for both future clinical practice and clinical trials. In clinical practice, AR apps could be used as screening tool for early AD or as an addition or replacement, even remotely applied, for neuropsychological assessment. For clinical trials, AR apps could be used as a clinical endpoint capable of assessing treatment response by monitoring cognitive and functional decline over time. The capacity of the app to offer a multi-level cognitive and functional evaluation of tasks relevant with various IADL, in a short period of time, represents indeed an interest for both screening and/or monitoring aspects. At-home tests can be performed to reduce the number of visits to a healthcare or research facility and facilitate early detection of AD in people living in remote areas. However, before an AR app can be used in clinical practice or clinical trials, the test-retest reliability should be improved, especially for iOS users, sensitivity to change should be measured, and our research should be replicated with a larger study sample. It is recommended that when the AR app is used as a clinical diagnostic tool, it will be used in a clinical setting with a standardized device. It is key that the app models should be tailored to distinguish HC from preAD participants.

Our study has several limitations that need to be taken into account. Apps are generally subjected to continued development, including changes in the design, functioning, or algorithms, which must be accounted for when designing studies or clinical trials. At the start of the current study, for example, the Android version of the AR app was less developed than the iOS version, leading to difficulties with the AR software. Another difficulty existed in the update to a second version of the app, including changes in the layout and instructions. Version number was therefore added as a covariate in all analyses. This update was not immediately available in all languages (initially only available in English, Spanish and German), which meant that not all participants who participated in the RADAR-AD study could use the AR app during a month. Following the delay, procedures continued as normal. Another limitation is that we did not exclude participants who used different devices during different at-home tests (*n* = 2), which could have influenced the results, especially of the test-retest reliability, although the sample size is small. Moreover, when testing remotely it is not possible to verify if participants performed the tests at home correctly and independently. On the other hand, these possibilities increase the generalizability of the results of this study, since the test environment or device of participants cannot be controlled with real-world monitoring. There were also 14 participants who did the first test (in the clinic) on a phone instead of an iPad 6 (2018), due to technical reasons. Removing these participants from the analysis resulted in similar findings for the discrimination of groups and correlations with clinical tests. The last limitation was that different versions of some neuropsychological tests were used with different raters in the different sites to accommodate the local language of each site. We therefore corrected for site in the analyses.

To our knowledge, this is the first study using an AR app in early AD. The AR app is feasible in the home setting and could distinguish HC from preAD participants, and HC from proAD participants. These results should encourage future studies with more participants and to specifically train the machine learning model to distinguish HC participants from preAD participants such that the accuracy of the classifier will increase.

## Methods

### Participants

**Fig. 5:**
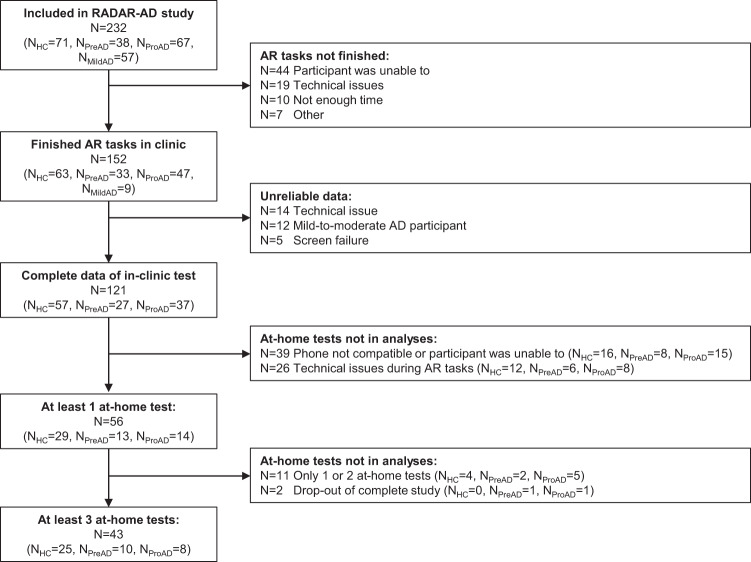
Flow diagram of the participant population included in this study. AR tasks were not finished because of the following reasons: (1) “Participant was unable to”: due to physical or cognitive problems the participant was unable to finish the test rounds successfully, (2) “Technical issues”: the app or device did not work as expected, (3) “Not enough time”: there was not enough time during the baseline visit to execute the tasks, (4) “Other reasons”: unspecified, unknown, or other reasons. AD Alzheimer’s disease, AR augmented reality, HC healthy control, RADAR-AD Remove Assessment of Disease and Relapse – Alzheimer’s Disease, preAD preclinical AD, proAD prodromal AD.

In this cross-sectional study, we included *N* = 121 participants (Fig. 5; Table 1 for demographic description) from the study Remote Assessment of Disease and Relapse – Alzheimer’s Disease (RADAR-AD). Inclusion criteria were community-dwelling adults older than 50 years of age, being able to communicate in the local language, and having a smartphone and a study partner available. Exclusion criteria were having a disease or disorder other than AD that could influence cognition and functioning in daily life. The participants were assigned to study groups, based on Mini-Mental State Examination (MMSE)^18^, Clinical Dementia Rating (CDR)^19^, and Aβ status (positive/negative), obtained via either Positron Emission Tomography (PET) or cerebrospinal fluid (CSF) analysis, defined by each site’s local procedures. The three study groups were defined as follows: healthy controls (MMSE > 27, CDR = 0, Aβ negative), preclinical AD (MMSE > 26, CDR = 0, Aβ positive), and prodromal AD (MMSE > 23, CDR = 0.5, Aβ positive), according to the NIA-AA criteria^20^. Participants with normal cognition were not asked about possible subjective cognitive decline and associated worries about their cognition. A fourth study group, the mild-to-moderate AD group from the RADAR-AD study^21^ was excluded from this research since the AR tasks were originally designed for participants with mild cognitive impairment and were therefore generally too difficult for the mild-to-moderate AD group. Participants were included from July 2020 to December 2022. Participants were recruited at 13 European study sites: Amsterdam Universitair Medische Centra (The Netherlands), King’s College London (United Kingdom), University of Oxford (United Kingdom), Karolinska Institutet in Stockholm (Sweden), Aristotle University of Thessaloniki (Greece), Carol Davila University of Medicine and Pharmacy in Bucharest (Romania), Ljubljana University Medical Centre (Slovenia), Faculdade de Medicina da Universidade de Lisboa (Portugal), IRCCS Istituto Centro San Giovanni di Dio Fatebenefratelli in Brescia (Italy), Zentralinstitut für Seelische Gesundheit Mannheim (Germany), Fundació ACE in Barcelona (Spain), Hôpitaux Universitaires de Genève in Geneva (Switzerland), and Centre for Age-Related Medicine in Stavanger (Norway). The appropriate ethical committees in the participating countries approved the study^22^ (Medisch Ethische Toetsingscommissie VUmc (2019.518), Drug Research Ethics Committee (CEIm) of Universitat International de Catalunya (MED-FACE-2020-07), Comitato Etico IRCCS Centro San Giovanni di Dio – Fatebenefratelli di Brescia, Commission cantonale d'éthique de la recherché (2022-00002), Comissão de Ética do Centro Académico de Medicina de Lisboa (388/19), London – West London & GTAC (Gene Therapy Advisory Committee) Research Ethics Committee (20/LO/0183), Ethics Committee II of the Ruprecht-Karls-University of Heidelberg (Medical Faculty Mannheim) (2020-508N), Regionale komiteer for medisinsk og helsefaglig forskningsetikk (98842), Swedish Ethical Review Authority (2020-03497), Ethics Committee of Medical Faculty of Aristotle University of Thessaloniki and Ethics Committee of Alzheimer Hellas (198/2018 AI). Participants and their legally authorized representatives (if appropriate) gave written informed consent before participating. The authors assert that all procedures contributing to this work comply with the ethical standards of the relevant national and institutional committees on human experimentation and with the Helsinki Declaration of 1975, as revised in 2008.

### Study procedures

Participants visited the memory clinic once for a baseline visit. During the visit, information was gathered on demographics, and cognition was assessed using a standard neuropsychological examination (see section “Neuropsychological assessment”). All HC, preAD, and proAD participants from the RADAR-AD study were asked to use the AR app if possible (sample size calculations for the entire study can be found in Muurling et al.^21^). The AR app was explained by a researcher. For the proAD group, study partners were asked to join the instructions, so that they could help during at-home tests if necessary. For the HC and preAD groups, presence of the study partner was optional. Each participant could practice the tasks until they fully understood the tasks without time limit, before starting the actual task. For all sites, the in-clinic test was completed on an iPad 6 (2018). Research staff was trained using a video training and study manual. During the in-clinic test, the participant was observed by the researcher, but the researcher did not provide any help. For participants who were able to use the app independently during the in-clinic assessment and who had a smartphone or tablet that was compatible with the AR app (both iOS and Android devices, Table 2), the app was installed on the participant’s smartphone, and the participants were asked to perform the test weekly at home for 8 weeks (Fig. 5). They received weekly email reminders to complete the tasks. These participants received a study manual containing detailed explanations on how to use the AR app at home, and answers to a set of frequently asked questions. For the proAD group, partners were asked to help with reminding the participant to use the AR app and starting the tasks in the app, but not with task completion. Bi-weekly phone calls with semi-structured interviews were performed by the local researcher to resolve potential technical issues. Research staff was available by phone or email in case any problems were encountered between the bi-weekly phone calls.

**Table 2:** At-home test characteristics per study group.

	Healthy control	Preclinical AD	Prodromal AD	*p* value
AR app tests at home, *n* (%)				0.09
0	16 (28%)	8 (30%)	15 (41%)	
<3	6 (11%)	3 (11%)	8 (22%)	
≥3	35 (61%)	16 (59%)	14 (38%)	
Using iOS, *n* (%)	23 (56%)	11 (58%)	12 (54%)	0.98
Using AR app version 2, *n* (%)	21 (51%)	15 (79%)	19 (86%)	0.008

Numbers show *n* (%). *P* values are from *χ*^2^ tests, uncorrected for any covariates.

*AD* Alzheimer’s disease, *AR* augmented reality, *iOS* iPhone Operating System.

### Neuropsychological assessment

The following neuropsychological test battery was administered during the baseline visit: a word list learning test, digit symbol substitution test (DSST)^23^, Rey complex figure^24^, verbal fluency^25^, and Boston naming test^26^. Different versions were used for the word list learning and Boston naming tests at different sites, with different number of words and different words, to ensure that each site used a validated version in their own language. For the word list learning test, Lisbon, Geneva, and Ljubljana sites used a 16-word version with 5 trials (Portuguese, French, and Slovenian, respectively), Stavanger site used a 10-word version with 3 trials (Norwegian), Barcelona site used a 12-word version with 4 trials (Spanish), and the other sites used a 15-word version with 5 trials in their local language. All sites used a delayed recall of 20 min. The participants from the Bucharest site (*N* = 2) were excluded from all analyses, because they used a different test battery (Alzheimer’s Disease Assessment Scale – Cognitive Subscale, which did not include a delayed recall). IADL was assessed using the caregiver-based Amsterdam IADL questionnaire, short version^4^.

### The AR app

**Fig. 6:**
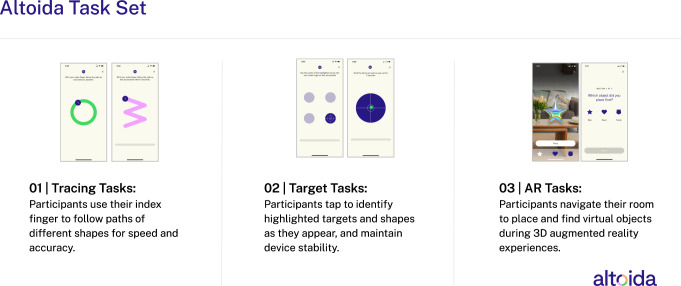
Examples of representative screenshots and task descriptions of the motor tasks (01 and 02) and AR tasks (03) in the Altoida app.

The AR app used from the Altoida Digital Biomarker Platform (the “Altoida app”) was developed by Altoida (Altoida Inc., Washington DC, USA) and consisted of one set of motor tasks and two AR tasks^27^. The motor tasks involved several different short exercises testing fine motor skills and reaction times to set reference values for motor skills, visual abilities, and reaction times (Fig. 6). The AR tasks simulated a complex IADL-like activity of a place-and-find task and a fire drill simulation, while faced with a distracting hearing exercise. During the place-and-find in the first AR task set, the participant had to walk around the room in which the assessment was conducted and place three virtual objects (e.g., a teddy bear, a star, and a heart). After several minutes, they had to find those objects in a random order (Fig. 6). The second task set was similar to the first, but this time, participants were given a defined set of actions to accomplish in a fire drill simulation, introducing additional memory and executive components. The participant first had to place three objects in the room (e.g., a fire alarm, a telephone, and important documents) and then find the objects in the correct order after a delay of several minutes (i.e., first hit the fire alarm, then ring the firemen using the telephone, and then save the important documents). More detailed information about the tasks can be found in Tarnanas et al.^28^ and Buegler et al.^27^. During the tasks, sensor information from the device was collected, including finger tapping data, movement, reaction time, correct responses, and navigational trajectory based on accelerometer and gyroscope data. The AR app was available in the local language of the participant, except for Norwegian. The participants from the Stavanger site (in Norway) used the AR app in Swedish instead, as the two languages are very similar.

Altoida updated their app on September 21, 2021, to a new version (version 2), meaning that one part of our study sample used version 1 and the other part used version 2. The tasks and model to calculate the digital score did not change, but the layout and instructions were improved. Moreover, two short motor tests were added to optimize the reference values for the AR tests. For details on the score output, see the “Clinical and digital outcome scores” section.

### Clinical and digital outcome scores

We calculated a clinical outcome score as a composite score of the total scores of each neuropsychological test used in the RADAR-AD study, including tasks measuring five cognitive domains (i.e., memory, executive functions, language, and complex attention). For the word list learning test, this was the percentage of the correct number of words that were remembered after a 20-min break (free recall). For the Boston naming test, this was the percentage of correctly named objects. The total verbal fluency score was the sum of words for three letters and animal fluency. The total score for the DSST was the total number of completed items after 90 s, with the number of incorrect items subtracted. For the Rey complex figure, the drawing score (copy) and recall score after 3 min were used. From all 6 total scores (word list recall, Boston naming test, verbal fluency, DSST, Rey drawing, and Rey recall scores), the z-score was calculated based on the control group (i.e., subtraction from the mean and divided by the standard deviation of the HC group), with higher *z*-scores indicating better cognition. The clinical cognitive composite score, hereafter called cognitive score, was the mean of the 6 z-scores. The A-IADL score was the total score calculated from the Amsterdam IADL questionnaire^4,29^.

The outcome of the motor and AR tasks combined was Altoida’s research algorithm Digital Neuro Signature® (DNS) – Mild Cognitive Impairment (MCI)^9^, hereafter called digital score. The digital score is a probability score ranging from 0 to 100, with higher scores indicating better performance and lower scores indicating a higher probability of having cognitive impairment. The score is based on a machine learning model trained to distinguish cognitively normal from cognitively impaired participants^27^, using the data from the touch screen, accelerometer, and gyroscope, and based on ground truth data from clinically validated cohorts.

### Statistical analyses

Groups were compared on demographic characteristics using ANOVAs or *χ*^2^ tests when appropriate. A sample size calculation is given in the design paper of the RADAR-AD study^21^.

To differentiate HCs from participants with early AD (primary aim), the three research groups were compared using a linear model, using the results of the first test in the clinic only, and correcting for the app version (1 or 2) and site. Age, sex, and years of education were not added as covariates, because the model to create the digital score already took these variables into account. Assumptions of normality and homogeneity of variances were confirmed using the Shapiro–Wilk test and Levene’s test, respectively. Sex-stratified results are presented in the Supplementary material (Supplementary Fig. 1). The model was repeated with the first at-home test to check for similar results between the at-home and in-clinic tests. For the association with clinical tests, Spearman’s correlation was used, since the assumption of linearity was not met after checking the residuals vs fitted plot of a linear model. Receiver operating characteristic (ROC) analyses were performed to test the accuracy of the in-clinic digital score, at-home digital score, cognitive score, and the A-IADL score to classify HC from preAD participants, and HC from proAD participants. The R-package “pROC” was used to calculate and plot the ROC curve and calculate the area under the curve (AUC). Since the group sizes were unbalanced, precision-recall (PR) curves were also computed, using the R-package “PRROC”.

To test the association between in-clinic and at-home tests (secondary aim), Spearman’s correlation was calculated between the first test in the clinic and the first test at home. A separate correlation was tested for each study group as well to test the associations per group.

To test any potential learning effects, a linear mixed-effects model was used, with the digital score as the dependent variable, the group, test number (as continuous variable), and group*test number interaction effect as independent variables, and random intercepts for each participant, corrected for phone type and AR app version. To avoid bias due to participants with better cognition completing more tests, only the first three at-home tests were used for this analysis. Moreover, test-retest reliability was tested using an intraclass correlation coefficient (ICC), calculated for the first three at-home tests (*k* = 3), based on a single measurement, absolute-agreement, two-way random effects model. ICC less than 0.5 was considered poor, between 0.5 and 0.75 considered moderate, between 0.75 and 0.9 good, and greater than 0.9 considered excellent reliability^30^. Since an effect of phone type was expected, separate ICCs were calculated for Android and iOS users.

To reduce biases due to external factors or day-to-day fluctuations, we repeated all analyses that used the first at-home test with the median of the first 3 at-home tests instead of the first at-home test only. Using this median reduces biases compared to when only 1 test was done, but lowers the number of participants, as not all participants did the test more than once at home. The results of these analyses will therefore be presented in the Supplementary material (Supplementary Figs. 3 and 4). We also repeated all analyses with a subgroup of participants who completed at least 3 at-home tests, to rule out effects due to selection bias. These results are also presented in the Supplementary material (Supplementary section “Repetition of tests with subgroup”).

A *p* value of <0.05 was considered statistically significant.

### Reporting summary

Further information on research design is available in the Nature Research Reporting Summary linked to this article.

### Supplementary information

Supplementary Material

Reporting Summary

## Data Availability

The data that support the findings of this study are available from the corresponding author upon reasonable request.

## Code availability

Analyses were performed in R (v4.2.1). The underlying code for this study is available from the corresponding author upon reasonable request.
