# Supplementary material for: Augmented reality versus standard tests to assess cognition and function in early Alzheimer’s disease
Source: NPJ Digit Med. 2023 Dec 18;6:234. doi: 10.1038/s41746-023-00978-6 (PMC10728213; doi:10.1038/s41746-023-00978-6)
Supplement: Supplementary file 1 — Supplementary Material [file 41746_2023_978_MOESM1_ESM.pdf]

Supplementary material

Discrimination of groups stratified for sex

We repeated the group comparisons, stratified for sex (Figure S1). For females, the proAD group scored significantly lower compared to the HC (β±SE=-27.2±6.7, p=0.003) with the in-clinic test, but not compared with the preAD group (β±SE=-4.6±9.1, p=0.61). The preAD group in females scored significantly lower compared to the HC group (β±SE=-22.6±7.1, p=0.003). For males, the proAD group scored significantly lower compared to the HC (β±SE=-32.3±6.8, p<0.001) and the preAD group (β±SE=-21.4±8.0, p=0.01), but the preAD group did not differ from the HC group (β±SE=-10.9±8.5, p=0.21). When comparing the three study groups on the digital score using the first at-home test only, stratified for sex, the differences between the HC and proAD groups disappeared for females (HC vs preAD: β±SE=-22.6±9.9, p=0.03, HC vs proAD: β±SE=-12.4±19.1, p=0.52, preAD vs proAD: β±SE=10.2±18.8, p=0.59). For males, the only difference found was between the HC and proAD groups (β±SE=-24.1±11.9, p=0.06, HC vs proAD: β±SE=-23.2±8.3, p=0.01, preAD vs proAD: β±SE=0.86±11.5, p=0.94).

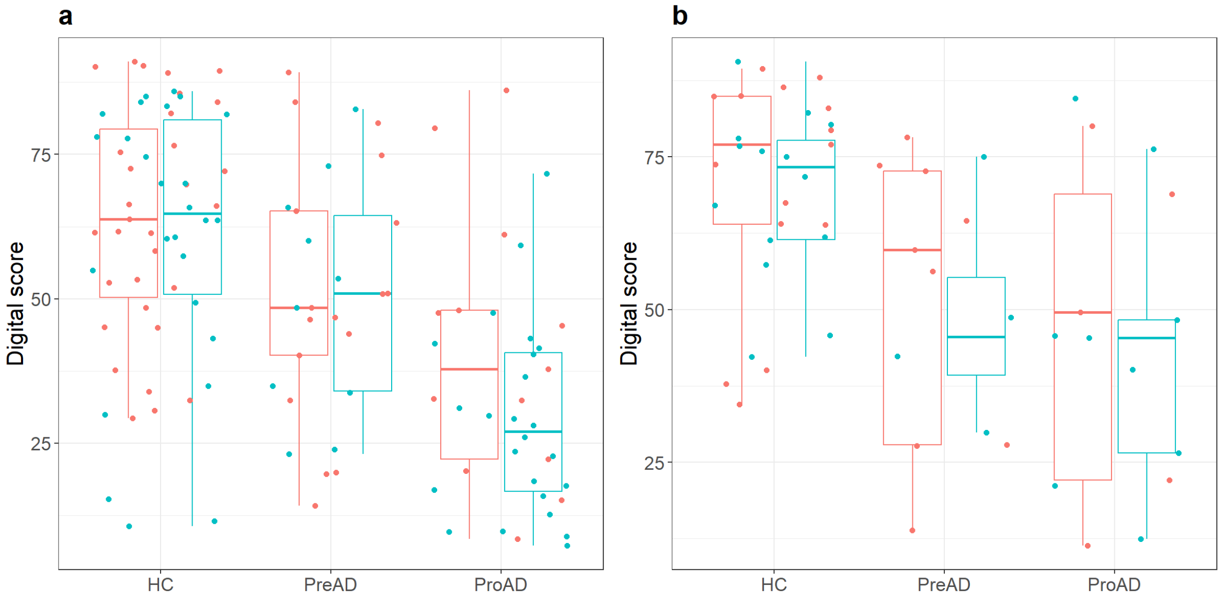

Supplementary Figure 1 *– a) In-clinic test. b) At-home test. Digital scores per group, stratified for sex. Each dot represents the score of one participant. The box represents the lower and upper quartiles with the center line the median, and the whiskers represent the minimum and maximum score. Red dots and boxplots show females, while blue dots and boxplots show males. Abbreviations: HC = healthy control, PreAD = Preclinical AD, ProAD = Prodromal AD.*

Precision-Recall curves

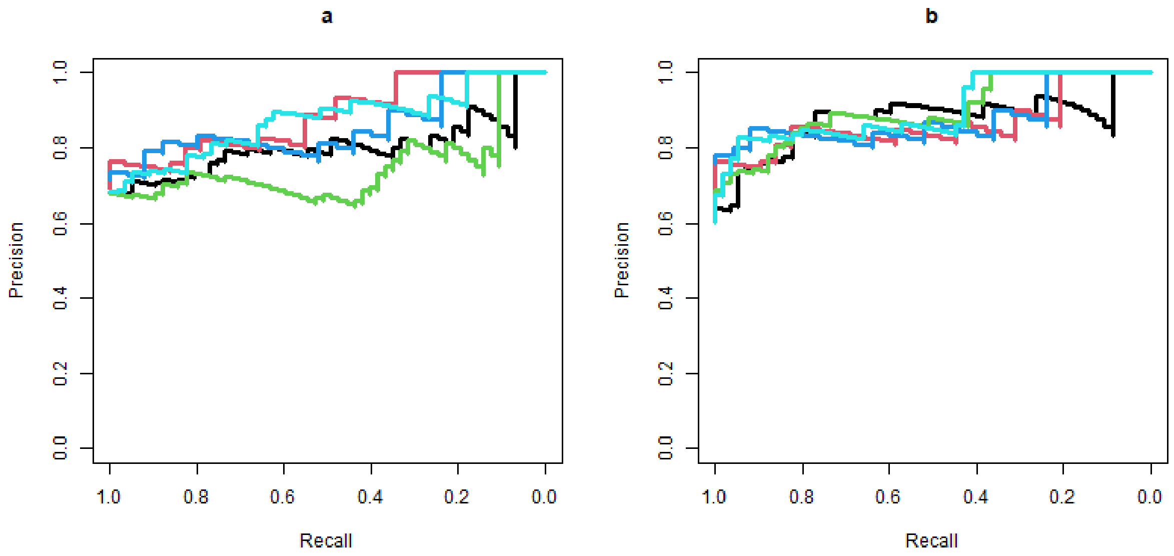

Supplementary Figure 2 *– PR-curves for the digital in-clinic, digital first at-home, digital median of first three at-home, cognitive, and A-IADL scores, to classify healthy controls relative to preAD (a), and healthy controls relative to proAD (b). Black line shows digital in-clinic test curve, red line shows digital first at-home test curve, dark blue line represents digital median of first three at-home tests curve, green lines shows cognitive score curve, and light blue line shows A-IADL score curve. Abbreviations: A-IADL = Amsterdam instrumental activities of daily living, HC = healthy control, PreAD = Preclinical AD, ProAD = Prodromal AD.*

Median of first three at-home tests

We repeated the tests mentioned in the statistical analysis paragraph with the median first three at-home tests, instead of the first at-home test only. When comparing the three study groups, effects flattened (HC vs preAD: β±SE=-14.5±7.1, p=0.05, HC vs proAD: β±SE=-13.3±9.5, p=0.16, preAD vs proAD: β±SE=1.2±9.6, p=0.90) (Figure S3A). The correlation between the in-clinic test and median of the first three at-home tests was rho=0.54 (p<0.001) (Figure S3B). When using three separate linear models per study group, the correlations for each group were similar (HC: rho=0.52, p=0.008; preAD: rho=0.49, p=0.15; proAD: rho=0.39, p=0.34). The discriminative power (Figure S4) of the median of the first three at-home tests was slightly worse than the first at-home test only (HC vs preAD: ROC-AUC=0.70 [0.47-0.92], PR-AUC = 0.85, HC vs proAD: ROC-AUC=0.70 [0.50-0.90], PR-AUC=0.88).

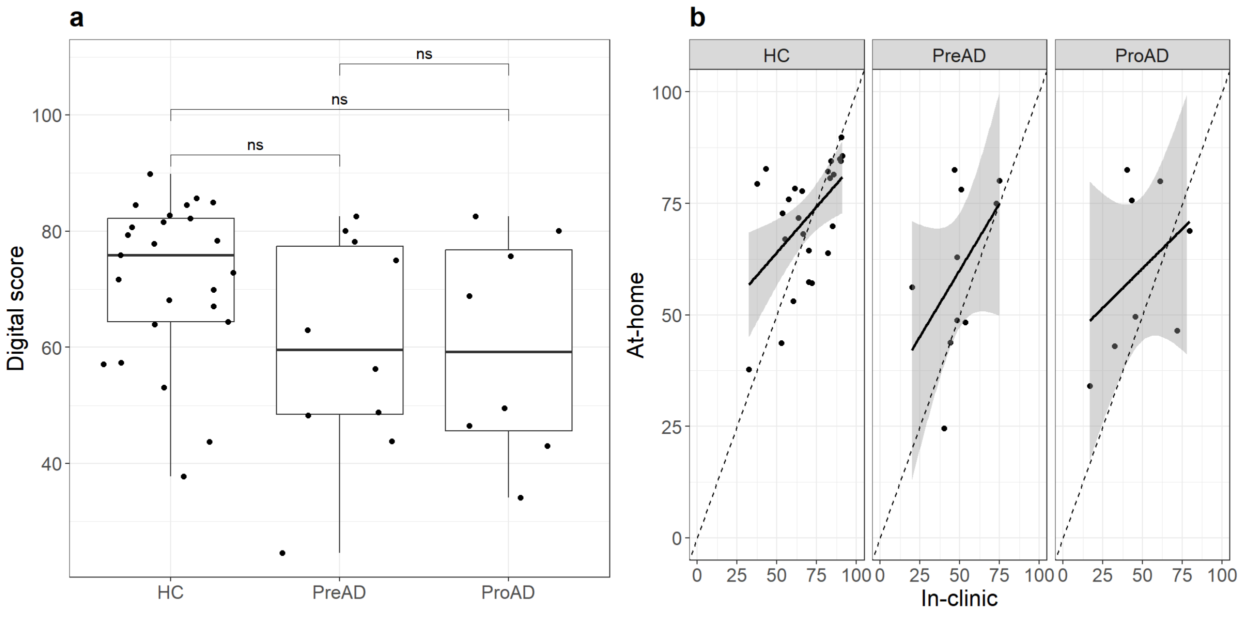

Supplementary Figure 3 *– a) Digital scores per group. Each dot represents the median digital score of the first three at-home tests of one participant. The box represents the lower and upper quartiles with the center line the median, and the whiskers represent the minimum and maximum score. Group differences were tested using a linear model, corrected for app version and site. Ns indicates not significant. b) Association between in-clinic and at-home tests. Each dot represents the median digital score of the first three at-home tests of one participant from one participant. The black dashed line is the rho=1 line. The black solid line represents the correlation line. Abbreviations: HC = healthy control, PreAD = Preclinical AD, ProAD = Prodromal AD.*

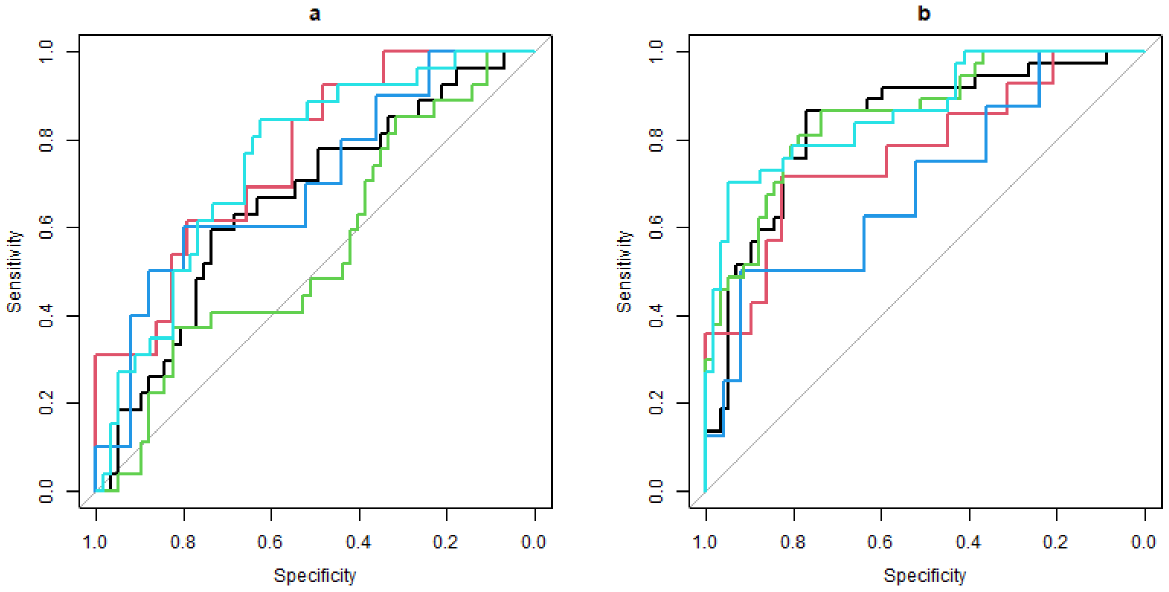

Supplementary Figure 4 *– ROC curves for the digital in-clinic, digital first at-home, digital median of first three at-home, cognitive, and A-IADL scores, to classify healthy controls relative to preAD (a), and*

healthy controls relative to proAD (b). Black line shows digital in-clinic test curve, red line shows digital first at-home test curve, dark blue line represents digital median of first three at-home tests curve, green lines shows cognitive score curve, and light blue line shows A-IADL score curve.. Abbreviations: A-IADL = Amsterdam instrumental activities of daily living, HC = healthy control, PreAD = Preclinical AD, ProAD = Prodromal AD.

Repetition of tests with subgroup

We repeated all analyses with the subgroup of participants who completed at least 3 tests at home (N=43), to rule out effects due to selection bias. When comparing the three study groups on the digital score resulting from the in-clinic AR task, the proAD group scored significantly lower compared to the HC (β±SE=-23.6±10.1, p=0.03). The preAD group also scored lower than the HC group (β±SE=-18.7±7.4, p=0.02). When comparing the three study groups on the digital score using the first at-home test only, findings were similar (HC vs preAD: β±SE=-20.2±7.0, p=0.007, HC vs proAD: β±SE=-10.3±9.3, p=0.28, preAD vs proAD: β±SE=9.8±9.5, p=0.31).

Correlation between the test in the clinic and the first test at home was rho=0.44 (p<0.001). When using three separate linear models per study group, the correlations for each group were similar (HC: rho=0.28, p=0.17; preAD: rho=0.56, p=0.10; proAD: rho=0.31, p=0.46).

The digital score was significantly associated with the cognitive score (Spearman's rho=0.37, p=0.01) and A-IADL score (Spearman's rho=0.27, p=0.08). For the classification of the proAD group, the digital in-clinic score (ROC-AUC=0.78 [0.59-0.97], PR-AUC = 0.92), digital at-home test (ROC-AUC=0.70 [0.46-0.93], PR-AUC = 0.87), and A-IADL score (ROC-AUC=0.80 [0.61-0.99], PR-AUC = 0.90) were as good as the cognitive score (ROC-AUC=0.77 [0.58-0.96], PR-AUC = 0.92). For classifying the preAD group, the digital in-clinic score (ROC-AUC=0.79 [0.62-0.96], PR-AUC = 0.91), digital at-home score (ROC-AUC=0.79 [0.62-0.96], PR-AUC = 0.91), and A-IADL score (ROC-AUC=0.72 [0.54-0.90], PR-AUC = 0.88) were superior to the cognitive score (ROC-AUC=0.49 [0.28-0.70], PR-AUC = 0.76).
