# Supplementary material for: Augmented reality versus standard tests to assess cognition and function in early Alzheimer’s disease
Source: NPJ Digit Med. 2023 Dec 18;6:234. doi: 10.1038/s41746-023-00978-6 (PMC10728213; doi:10.1038/s41746-023-00978-6)
Supplement: Supplementary file 2 — Reporting Summary [file 41746_2023_978_MOESM2_ESM.pdf]

nature portfolio

Corresponding author(s): *Double-anonymous peer review submissions: write DAPR and your manuscript number here instead of author names.*

Last updated by author(s): *YYYY-MM-DD*

Reporting Summary

Nature Portfolio wishes to improve the reproducibility of the work that we publish. This form provides structure for consistency and transparency in reporting. For further information on Nature Portfolio policies, see our Editorial Policies and the Editorial Policy Checklist.

Statistics

For all statistical analyses, confirm that the following items are present in the figure legend, table legend, main text, or Methods section.

n/a	Confirmed	
☐	☒	The exact sample size (*n*) for each experimental group/condition, given as a discrete number and unit of measurement
☐	☒	A statement on whether measurements were taken from distinct samples or whether the same sample was measured repeatedly
☐	☒	The statistical test(s) used AND whether they are one- or two-sided<br>*Only common tests should be described solely by name; describe more complex techniques in the Methods section.*
☐	☒	A description of all covariates tested
☐	☒	A description of any assumptions or corrections, such as tests of normality and adjustment for multiple comparisons
☐	☒	A full description of the statistical parameters including central tendency (e.g. means) or other basic estimates (e.g. regression coefficient) AND variation (e.g. standard deviation) or associated estimates of uncertainty (e.g. confidence intervals)
☒	☐	For null hypothesis testing, the test statistic (e.g. *F*, *t*, *r*) with confidence intervals, effect sizes, degrees of freedom and *P* value noted<br>*Give P values as exact values whenever suitable.*
☒	☐	For Bayesian analysis, information on the choice of priors and Markov chain Monte Carlo settings
☒	☐	For hierarchical and complex designs, identification of the appropriate level for tests and full reporting of outcomes
☐	☒	Estimates of effect sizes (e.g. Cohen's *d*, Pearson's *r*), indicating how they were calculated

Our web collection on statistics for biologists contains articles on many of the points above.

Software and code

Policy information about availability of computer code

Data collection	The AR app used from the Altoida Digital Biomarker Platform (the 'Altoida app') was developed by Altoida (Altoida Inc., Washington DC, USA)
Data analysis	Analyses were performed in R (v4.2.1)

For manuscripts utilizing custom algorithms or software that are central to the research but not yet described in published literature, software must be made available to editors and reviewers. We strongly encourage code deposition in a community repository (e.g. GitHub). See the Nature Portfolio guidelines for submitting code & software for further information.

Data

Policy information about availability of data

All manuscripts must include a data availability statement. This statement should provide the following information, where applicable:

- Accession codes, unique identifiers, or web links for publicly available datasets
- A description of any restrictions on data availability
- For clinical datasets or third party data, please ensure that the statement adheres to our policy

The data that support the findings of this study are available from the corresponding author upon reasonable request.

Research involving human participants, their data, or biological material

Policy information about studies with human participants or human data. See also policy information about sex, gender (identity/presentation), and sexual orientation and race, ethnicity and racism.

Reporting on sex and gender	We collected self-reported sex, and used this for sex-stratified analyses.
Reporting on race, ethnicity, or other socially relevant groupings	N/A
Population characteristics	We used the covariates age, sex and years of education. Population characteristics are shown in Table 1.
Recruitment	Participants were recruited from memory clinics or ongoing observational studies.
Ethics oversight	Participants were recruited at 13 European study sites: Amsterdam Universitair Medische Centra (The Netherlands), King's College London (United Kingdom), University of Oxford (United Kingdom), Karolinska Institutet in Stockholm (Sweden), Aristotle University of Thessaloniki (Greece), Carol Davila University of Medicine and Pharmacy in Bucharest (Romania), Ljubljana University Medical Centre (Slovenia), Faculdade de Medicina da Universidade de Lisboa (Portugal), IRCCS Istituto Centro San Giovanni di Dio Fatebenefratelli in Brescia (Italy), Zentralinstitut für Seelische Gesundheit Mannheim (Germany), Fundació ACE in Barcelona (Spain), Hôpitaux Universitaires de Genève in Geneva (Switzerland), and Centre for Age-Related Medicine in Stavanger (Norway). The appropriate ethical committees in the participating countries approved the study (see Muurling et al., 2023).

Note that full information on the approval of the study protocol must also be provided in the manuscript.

Field-specific reporting

Please select the one below that is the best fit for your research. If you are not sure, read the appropriate sections before making your selection.

☒ Life sciences ☐ Behavioural & social sciences ☐ Ecological, evolutionary & environmental sciences

For a reference copy of the document with all sections, see nature.com/documents/nr-reporting-summary-flat.pdf

Life sciences study design

All studies must disclose on these points even when the disclosure is negative.

Sample size	Sample size calculation for the study is described in Muurling, M., de Boer, C., Kozak, R., Religa, D., Koychev, I., Verheij, H., ... & Visser, P. J. (2021). Remote monitoring technologies in Alzheimer's disease: design of the RADAR-AD study. Alzheimer's research & therapy, 13(1), 89.
Data exclusions	We excluded the participants that indicated technical issues, as shown in the flow diagram (Figure 5).
Replication	Study is not replicated yet.
Randomization	This is not a clinical trials and therefore randomization is not applicable.
Blinding	This is not a clinical trials and therefore blinding is not applicable.

Reporting for specific materials, systems and methods

We require information from authors about some types of materials, experimental systems and methods used in many studies. Here, indicate whether each material, system or method listed is relevant to your study. If you are not sure if a list item applies to your research, read the appropriate section before selecting a response.

Materials & experimental systems

n/a	Involved in the study	
☒	☐	Antibodies
☒	☐	Eukaryotic cell lines
☒	☐	Palaeontology and archaeology
☒	☐	Animals and other organisms
☐	☒	Clinical data
☒	☐	Dual use research of concern
☒	☐	Plants

Methods

n/a	Involved in the study	
☒	☐	ChIP-seq
☒	☐	Flow cytometry
☒	☐	MRI-based neuroimaging

Clinical data

Policy information about clinical studies

All manuscripts should comply with the ICMJE guidelines for publication of clinical research and a completed CONSORT checklist must be included with all submissions.

Clinical trial registration	This study is no clinical trial and therefore not registered.
Study protocol	Described in Muurling, M., de Boer, C., Kozak, R., Religa, D., Koychev, I., Verheij, H., ... & Visser, P. J. (2021). Remote monitoring technologies in Alzheimer’s disease: design of the RADAR-AD study. Alzheimer's research & therapy, 13(1), 89.
Data collection	Participants were recruited at 13 European study sites: Amsterdam Universitair Medische Centra (The Netherlands), King’s College London (United Kingdom), University of Oxford (United Kingdom), Karolinska Institutet in Stockholm (Sweden), Aristotle University of Thessaloniki (Greece), Carol Davila University of Medicine and Pharmacy in Bucharest (Romania), Ljubljana University Medical Centre (Slovenia), Faculdade de Medicina da Universidade de Lisboa (Portugal), IRCCS Istituto Centro San Giovanni di Dio Fatebenefratelli in Brescia (Italy), Zentralinstitut für Seelische Gesundheit Mannheim (Germany), Fundació ACE in Barcelona (Spain), Hôpitaux Universitaires de Genève in Geneva (Switzerland), and Centre for Age-Related Medicine in Stavanger (Norway). Data was collected from July 2020 to January 2023.
Outcomes	Please see our publication above (Muurling et al., 2021).

Plants

Seed stocks	N/A
Novel plant genotypes	N/A
Authentication	N/A
